# Influence of magnesium on the structure and hydration of perfluorooctanoate micelles: comparison with sodium salt

**DOI:** 10.1039/d6ra02082h

**Published:** 2026-08-13

**Authors:** Njelama Sanga, Lutz Ahrens, Gregory N. Smith, Adrian R. Rennie

**Affiliations:** a Macromolecular Chemistry, Department of Chemistry –Ångström, Uppsala University Box 539 75120 Uppsala Sweden njelama.sanga@kemi.uu.se adrian.rennie@kemi.uu.se; b Department of Aquatic Sciences and Assessment, Swedish University of Agricultural Sciences (SLU) Box 7050 75007 Uppsala Sweden; c ISIS Neutron and Muon Source, Science and Technology Facilities Council, Rutherford Appleton Laboratory Didcot OX11 0QX UK

## Abstract

The influence of counterions such as magnesium on the assembly and dispersion of perfluorooctanoate, which is an environmentally persistent fluorocarbon surfactant, is important for its transport in the environment. In this study, the structure and hydration of magnesium perfluorooctanoate micelles were investigated as a function of temperature using small-angle scattering techniques. Multiple water contrasts using isotopes for neutron studies, complemented by X-ray scattering, provide data for fitting detailed structural models. The magnesium perfluorooctanoate formed elongated rod-like micelles with a dense core of 1.86 g cm^−3^ and a hydrated shell containing ∼10 water molecules per perfluorooctanoate molecule. The length of the micelles increased at lower temperatures while the number of water molecules per head group remained almost constant. A pronounced isotope effect was observed at low temperatures: micelles were approximately five times longer in D_2_O compared to H_2_O. In the presence of added salt, magnesium perfluorooctanoate precipitated and remained insoluble at elevated temperatures. Sodium perfluorooctanoate formed shorter ellipsoidal micelles with a length of 18 Å and a hydrated shell containing approximately seven water molecules, with a less dense core of 1.64 g cm^−3^. The presence of counterions such as magnesium and sodium influences the transport and dispersion of perfluorooctanoate in the environment. The results provide valuable insight into remediation strategies, including salt-induced precipitation to concentrate the surfactant before subsequent degradation.

## Abstract 2

The influence of counterions such as magnesium on the assembly and dispersion of perfluorooctanoate, which is an environmentally persistent fluorocarbon surfactant, is important for understanding its transport in the environment.

## Graphical abstract

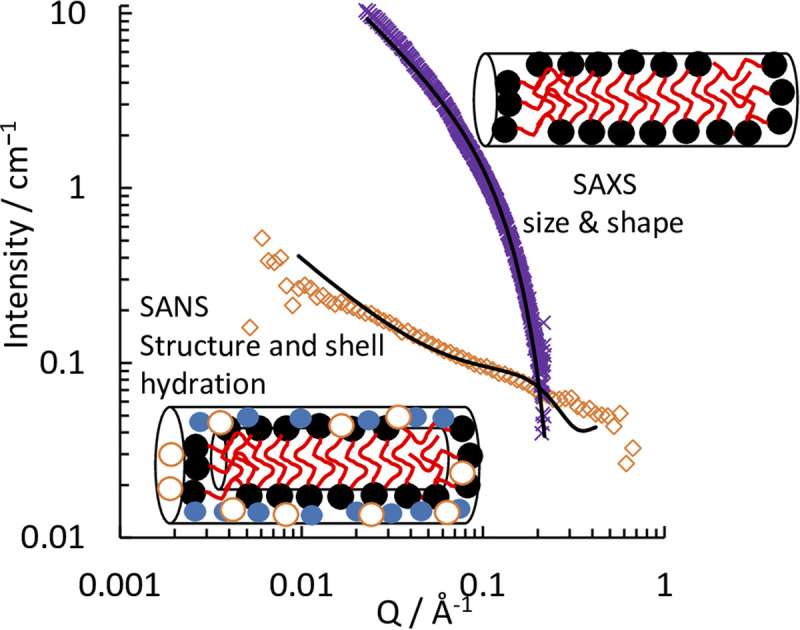

## Introduction

The self-assembly of perfluorooctanoate influences its transport in the environment. Above their critical micelle concentration (CMC), perfluorooctanoate surfactants form aggregates that diffuse more slowly than the individual molecules, but as the micelles transport more molecules, they can provide more efficient transport. The rate of diffusion depends on the size and shape of the micelles or other aggregates.^1^ Counterions strongly influence the self-assembly and dispersion of perfluorooctanoate by altering the molecular packing and also by providing effective screening of electrostatic micellar interactions. For example, ammonium^2^ and sodium perfluorooctanoate micelles^3^ form slightly elongated micelles that have been described as cylinders or prolate ellipsoids, while caesium perfluorooctanoate molecules pack in less curved assemblies, forming oblate ellipsoid micelles.^4^ One previous study approximated the shape of sodium perfluorooctanoate micelles to spheres,^5^ but the overall size was inconsistent with the molecular dimensions, and so presumably, the spheres represent an approximation to other shapes. However, these various studies did not explicitly describe the core and the hydrated shell, and they did not explain how the micelles assemble.

The divalent counterion Mg^2+^ influences the micellar structure in a manner different from that of the monovalent counterions (Na^+^, NH_4_^+^, Cs^+^). Previous small-angle X-ray scattering studies^6^ have shown that magnesium perfluorooctanoate forms elongated micelles that are much longer than those observed for the monovalent salts (Li^+^, Na^+^, NH_4_^+^). The reduced curvature caused by the Mg^2+^ ions favours closer packing and assembly as elongated cylinders. The critical micelle concentration was determined by measurements of solution conductivity to be 7.8 mmol dm^−3^ for the magnesium salt,^6^ which is about three times lower than that for the monovalent salts. That study also identified that both concentration and temperature alter the assembly of the surfactants, with more elongated micelles observed at lower temperatures and for higher surfactant concentrations.

However, small-angle X-ray scattering alone cannot fully resolve the molecular arrangement and hydration of magnesium perfluorooctanoate micelles. The combination of X-ray scattering and neutron scattering measurements on the same system allows structural details to be determined because of the different contrast of the various components, particularly when exploiting the different scattering power of neutrons for normal hydrogen and deuterium.^7^ For example, a mixture of heavy water (D_2_O) and normal water (H_2_O) can be prepared that matches the scattering from the fluoroalkyl chains. This can be seen from the values of the scattering lengths listed in Table 1. The contrasts for X-rays, which are also different, are also shown. In this way, the arrangement of the hydrated ionic headgroups can be investigated. As will be described below, it is important to ascertain the extent of any effect of different solvation, dissociation and hydrogen bonding of the two isotopically different solvents. In some surfactant systems, significant effects have been identified previously. As examples, for some non-ionic surfactants with glucoside^8,9^ and ethylene oxide^10^ hydrophilic moieties, large differences in solvation are seen near phase boundaries. The critical micelle concentration for dodecyldimethylphosphine^11,12^ was about 25% lower in D_2_O than in H_2_O. An enthalpy of micellization that was 30% higher in D_2_O than in H_2_O was found for potassium decanoate^13^ at 25 °C. It is therefore clearly important to verify the extent and origin of such effects.

**Table 1:** Neutron and X-ray scattering lengths^7^ for materials studied

Moiety	Neutron scattering length Σ*b*/fm	X-ray scattering length Σ*b*/fm
C_7_F_15_	131.33	503.9
Mg^2+^	5.38	34.4
Na^+^	3.63	31.4
H_2_O	−1.68	28.3
D_2_O	19.15	28.3
COO^−^	18.25	62.3

The molecular volumes of the solvated carboxylate headgroups, counterions and water are important parameters with regard to the micellar packing and hydration. The approach in this study has been to determine the size and aggregation number of micelles and the packing density of the individual surfactant moieties by analysing scattering measurements acquired using absolute scales. This contrasts with previous studies of related systems that have used estimated values or assumed densities (Table S1 in SI) to constrain fits to data. The previous values will be compared with the new scattering measurements.

This study combines small-angle X-ray scattering with small-angle neutron scattering using multiple water contrasts (D_2_O, H_2_O and fluorocarbon-matched water) to determine micellar core density and the carboxylate headgroup hydration. In addition, the effects of temperature and added salt were also investigated, and the results were compared directly with those of the monovalent sodium perfluorooctanoate salt.

## Experimental

### Surfactants

Perfluorooctanoic acid (C_7_F_15_COOH, purity: 95%) and sodium hydroxide (NaOH, 98% pellets) were purchased from Sigma-Aldrich, and magnesium hydroxide (Mg(OH)_2_, 95–100%) was purchased from Fluka. Solutions were made using deionized water with a resistivity of 18.2 MΩ cm. The salts were prepared by neutralization of the acid with the appropriate metal hydroxide, as described previously.^6^ Electrophoresis measurements were made using a Malvern Zetasizer Pro as described in that paper. Further details about sample preparation for SANS and SAXS are provided in the SI.

### Small-angle neutron scattering measurements

The small-angle neutron scattering experiments were performed on the Larmor instrument^14^ at the ISIS Neutron and Muon Source, Rutherford Appleton Laboratory, Didcot, United Kingdom. Larmor is a fixed-configuration, time-of-flight SANS instrument with a sample-to-detector distance of 4 m and a wavelength, *λ*, range of 0.9 < *λ* < 12.8 Å. The samples for magnesium perfluorooctanoate and sodium perfluoro-octanoate were prepared by dilution from stock solutions of 150 mmol dm^−3^ and 300 mmol dm^−3^, respectively. Sample solutions were injected with a syringe into fused quartz cells of 2 mm thickness for samples in D_2_O and of 1 mm thickness for samples in H_2_O and mixtures of H_2_O with D_2_O. The samples were measured over a temperature range of 10–50 °C. A glycol bath was used to decrease the temperature, and dry air was circulated through the sample holder to prevent condensation. The experiment used three different water contrasts: 100% D_2_O, 64%/36% D_2_O/H_2_O (fluorocarbon matched water, FMW), and 100% H_2_O. Sample transmission and scattering measurements were used to give reduced data corrected for background scattering using Mantid software version 6.10.0, with the ISIS SANS user interface.^15^ Data were placed on an absolute scale by measuring the scattering from a mixture of hydrogenous and deuterated polystyrene used as a secondary standard with known radius of gyration and scattering cross section in the manner described by Wignall and Bates.^16^ The data are presented as plots of intensity of scattered radiation *vs.* the scattering vector *Q* = (4π/*λ*) sin(*θ*/2), where *λ* is the wavelength and *θ* is the scattering angle. For this configuration on Larmor measurements are in the range 0.005 < *Q* < 0.7 Å^−1^. In scattering experiments, the vector *Q* represents the momentum transfer between the incoming wave and the scattered wave.

### Small-angle X-ray scattering measurements

Small-angle X-ray scattering measurements were performed on the Xeuss 2.0 Q-Xoom (Xenocs, Grenoble, France) instrument at the Department of Medicinal Chemistry, Uppsala University, as described in previous work.^6^ Samples of 100 mmol dm^−3^ magnesium perfluorooctanoate were prepared in D_2_O and H_2_O contrasts and sealed into 1 mm diameter borosilicate glass capillaries (from Hilgenberg). The samples were measured in the temperature range of 10–50 °C using a Peltier-controlled stage and were maintained under vacuum during measurements.

### Interpretation of small-angle scattering results

The approach to data analysis and model fitting used in this work is slightly different from that in most other studies of micelles. The difference arises from the fitting strategy, in which the core density is constrained to be identical for all contrasts, allowing the calculation of the scattering length densities of the components of the shell, and this is described in more detail below. The analysis followed well-established procedures, specifically to fit form factors that account for shapes appropriate to micelles that are available in the SasView software,^17,18^ exploiting combined fits to data sets measured with different scattering contrasts. Where appropriate, these can be combined with structure factor functions accounting for the interaction of micelles.

For the magnesium salt, inspection of the scattering data indicated the presence of elongated micelles with no additional structural features, suggesting that there are no inter-micellar interactions. Dissolved divalent magnesium ions provide stronger electrostatic screening, resulting in a shorter Debye length and much weaker positional correlation of micelles compared to sodium ions. Accordingly, the data were modelled as core–shell cylinders, with multiple data sets measured at different water contrasts fitted simultaneously using a single model in a combined least squares minimisation. The fitting was constrained to ensure that the model remained physically realistic, that all the scattering data were determined on an absolute scale of intensity and accounted for all material. Furthermore, simultaneous fitting of two data sets provided sufficient structural information to reliably determine the model parameters. At higher concentrations, the micelles became more elongated, consistent with an increased mean separation.

The data for sodium perfluorooctanoate were modelled as core–shell ellipsoids with interactions and the structure factor *S*(*Q*) was fitted considering the volume fraction, charge and the electrostatic screening due to the dissolved ions. The models consisted of core regions corresponding to fluoroalkyl chain assemblies without water and surrounding hydrated headgroups and counterions in a shell. A general description of the construction of such model functions for scattering has been provided by Pedersen.^19^ The scattering function for a core–shell ellipsoid of revolution has been stated explicitly elsewhere.^20^

### Strategies for fitting small-angle scattering data

**Fig. 1:**
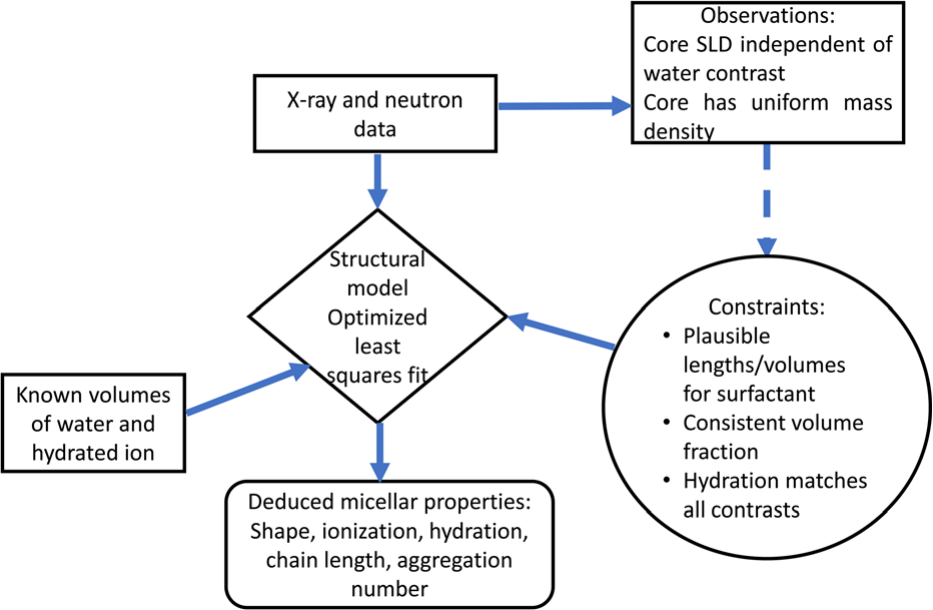
Flow chart summarising the model fitting strategy used to determine the structural model of magnesium perfluorooctanoate micelles using an optimised least square fit.

The small-angle X-ray and neutron data show that the scattering length density of the core does not depend on the water contrast and thus contains no water. The core mass density is determined from the data and is constant. The values for the core scattering length density, *ρ*_c_, were allowed to vary but were constrained so that the value for neutron scattering data corresponded to that for X-rays for fluorocarbon material with the same mass density. These observations form the basis for a set of constraints used to obtain a physically realistic structural model of magnesium perfluorooctanoate micelles. A key constraint is a realistic molecular length of the perfluorooctanoate molecule. Previous studies suggested a length of 10.4 Å for a hydrocarbon molecule,^21^ but this value does not include the headgroup. Ijima *et al.*^4^ proposed a different length of 13 Å, which accounts for both the extended fluorocarbon chain and the headgroup, and this value was adopted as an initial value. Additional constraints included maintaining a consistent micelle volume fraction across all contrasts, as this parameter considerably impacts the observed intensity. The hydration was constrained to be the same for all the contrasts. Knowledge of the known volumes of water and the hydrated counterion is therefore essential for constructing the complete structural model. Micellar properties were subsequently deduced using these constraints with the appropriate model. The overall data analysis and model-fitting strategy are summarised schematically in Fig. 1.

The present analysis exploits the possibility of deriving the aggregation number, *N*_agg_, directly from the product of the core volume, *V*_c_, and the fitted scattering length density of that region, *ρ*_c_, divided by the total scattering length of the fluoroalkyl chain, *b*_fc_:

1 *N*_agg_ = *V*_c_*ρ*_c_/*b*_fc_.

The composition of the hydrated ‘shell’ could then be determined from knowledge of the aggregation number, the scattering length of the shell per molecule, and the known stoichiometry of the fluorocarbon surfactant. This information, together with the degree of ionic dissociation, *b*_h_, which provides the scattering length of the surfactant headgroups, was used to calculate the hydration. The remaining scattering length in the shell region was attributed to water molecules appropriate to the measurement contrast. The number of water molecules per surfactant headgroup, *N*_w_, is then given by:

2 *N*_w_ = (*V*_s_*ρ*_s_ − *N*_agg_*b*_h_)/(*N*_agg_*b*_w_)

where *V*_s_ is the volume of the shell, *ρ*_s_ is the shell scattering length density, *N*_agg_ is the aggregation number, *b*_h_ is the scattering length of the headgroup, and *b*_w_ is the scattering length of the water molecules for a given contrast. Further details of these relations are provided in the SI, Section S2 and eqn (S1) to (S4).

The models fitted using SasView^17^ were assessed with the reduced chi-squared parameter, *χ*_R_^2^, as described in the SI, Section S4. Any fits that did not provide consistent values for *N*_w_ were rejected, and alternative starting parameters were used. This would typically involve manual variation of the thickness of the hydrated shell. It should be noted that, as for the core, the overall density of the shell is not constrained in this fitting procedure but is instead determined directly from the data, allowing comparison with other studies and measurements. This approach requires that at least one scattering contrast has significant contributions to the measured intensity arising from the shell that is different from another contrast.

## Results and discussion

This section begins with an overview of the effects of isotope substitution observed at lower temperatures for magnesium perfluorooctanoate in D_2_O and at room temperature for sodium perfluorooctanoate, as shown by small-angle X-ray scattering measurements. These effects are important because combined fitting of models to neutron data measured with solutions containing different water isotopes at these temperatures is not possible due to the considerable difference in micellar size. Results concerning the solubility of perfluorooctanoate salts in the presence of added salts are presented first. This is followed by describing the micellar shape, dimensions and hydration of magnesium perfluorooctanoate micelles using data from small-angle neutron and small-angle X-ray scattering measurements, under conditions where isotope effects are small. These results are then compared with those of the monovalent salt sodium perfluorooctanoate in the following section. Finally, the effects of temperature are described together with a general discussion of the results.

### Isotope effects

**Fig. 2:**
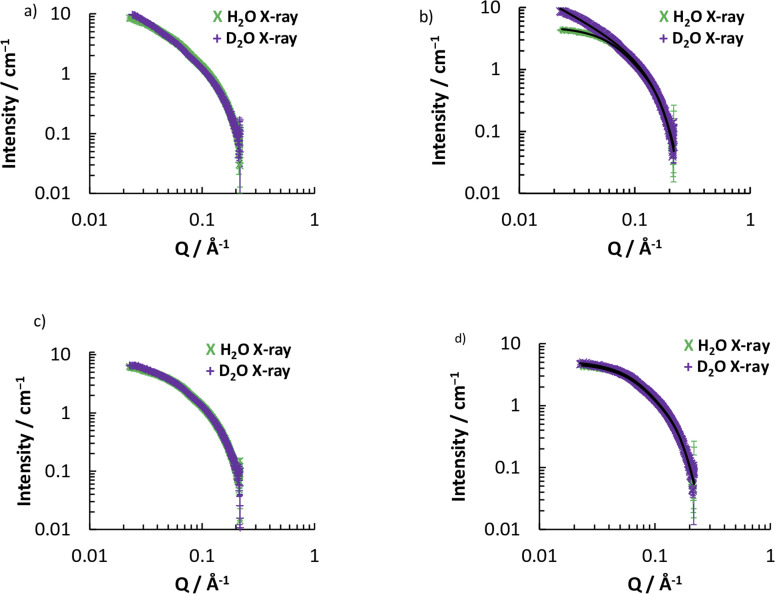
Small-angle X-ray scattering data for 100 mmol dm^−3^ magnesium perfluorooctanoate measured at (a) 10 °C, (b) 15 °C, (c) 25 °C, and (d) 50 °C in D_2_O and H_2_O. The continuous lines represent fits of models for a cylinder.

**Fig. 3:**
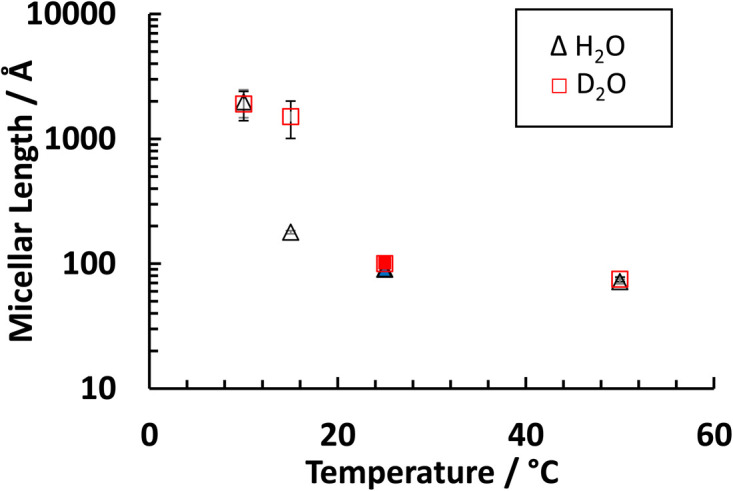
Length of magnesium perfluorooctanoate micelles as a function of temperature in H_2_O and D_2_O. The fitted values are taken from Table S2 in the SI. As the radii do not change significantly, the aggregation number shows a similar trend with temperature as the length. The filled symbols indicate the results from repeated measurements after reheating to 25 °C, which are indistinguishable from the initial data.

Small-angle X-ray scattering data for 100 mmol dm^−3^ magnesium perfluorooctanoate at 10 °C and 15 °C are shown together with corresponding model fits in Fig. 2. This experimental method was sensitive primarily to the density of the core of the micelles and not to hydration of the ionic groups because of the high electron density of the fluoroalkyl chains. At 25 °C and 50 °C (Fig. 2), no differences were observed in the scattering in H_2_O and D_2_O water contrasts. The same physical model was fitted to all data sets, and it was observed that the elongation decreased with decreasing temperatures in both contrasts. The parameters for micellar size obtained from the fits to the individual data sets are shown in Table S2, and the corresponding Kratky plots (*I*(*Q*) *Q*^2^*vs. Q*) for these data are shown in Fig. S1 in the SI. This confirms behaviour observed in previous X-ray studies on magnesium perfluorooctanoate salts at elevated temperatures.^6^ However, at lower temperatures, particularly at 15 °C, the scattering showed marked differences between the samples in D_2_O and H_2_O. The intensity in D_2_O was higher than in H_2_O, and the shape of the scattering curve was quite different, suggesting changes in micellar length due to isotope effects. It appears that substitution of hydrogen with deuterium in the water has an analogous effect to that of lowering temperature. However, when the temperature is lowered further from 15 °C to 10 °C, the effect of temperature dominates over the isotope effect. On subsequent reheating of the samples to 25 °C, data corresponding to the initial measurements with no differences were measured. The repeatable changes of micellar length can be seen in Fig. 3, where the length of the micelles in each solvent is shown as a function of temperature.

The zeta potential of sodium perfluorooctanoate in both H_2_O and D_2_O at 25 °C was 54 ± 10 mV. These effects likely arose because the changes in hydrogen bonding and water density between H_2_O and D_2_O were proportionally larger at the low temperatures, thereby influencing micellar aggregation number and anisotropy.^13^

**Fig. 4:**
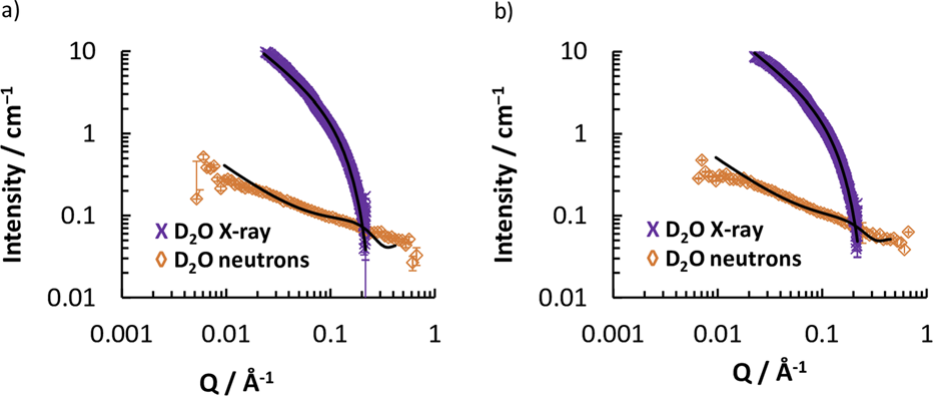
Small-angle neutron scattering and X-ray scattering of magnesium perfluorooctanoate at (a) 10 °C and (b) 15 °C, both measured in D_2_O, showing the fit when only these data are considered. The parameters are shown in Table S3 in the SI.

Further measurements were made with small-angle neutron scattering, as neutrons are more sensitive to micellar regions with different scattering length densities, and thus to hydration of the headgroups. At 15 °C, simultaneous fitting of all contrasts was not possible. Instead, the neutron data in D_2_O were fitted together with just the X-ray scattering data in D_2_O (Fig. 4), as described in the following section.

Some effects that are comparable with these observations for magnesium perfluorooctanoate when substituting hydrogen isotopes in water have been described in previous studies on ammonium perfluorooctanoate^22^ and caesium perfluorooctanoate,^23^ as discussed further below.

### Observations on the solubility of perfluorooctanoate salts

The effects of added salts (Na^+^, Mg^2+^, and Ca^2+^) on the solubility of perfluorooctanoate salts were assessed by visual observation. In the presence of sodium chloride, sodium perfluorooctanoate remained soluble up to 0.2 mol dm^−3^ with no precipitation observed. In contrast, the addition of magnesium chloride hexahydrate reduced the solubility of 100 mmol dm^−3^ magnesium perfluorooctanoate. Increasing concentrations of added salt led to progressive precipitation of the surfactant. Heating the samples to 50 °C did not increase the solubility of the magnesium perfluorooctanoate as the surfactant remained insoluble (Fig. S2). Fig. S3 in the SI shows the phase diagram of magnesium perfluorooctanoate as a function of concentration and temperature without any added salt. The diagram shows that the compound is insoluble below 10 °C and that micellization occurs above 8 mmol dm^−3^. Calcium perfluorooctanoate salt was found to be insoluble and did not form a measurable dispersion even at higher temperatures.

### Magnesium perfluorooctanoate – micellar shape and dimensions

**Fig. 5:**
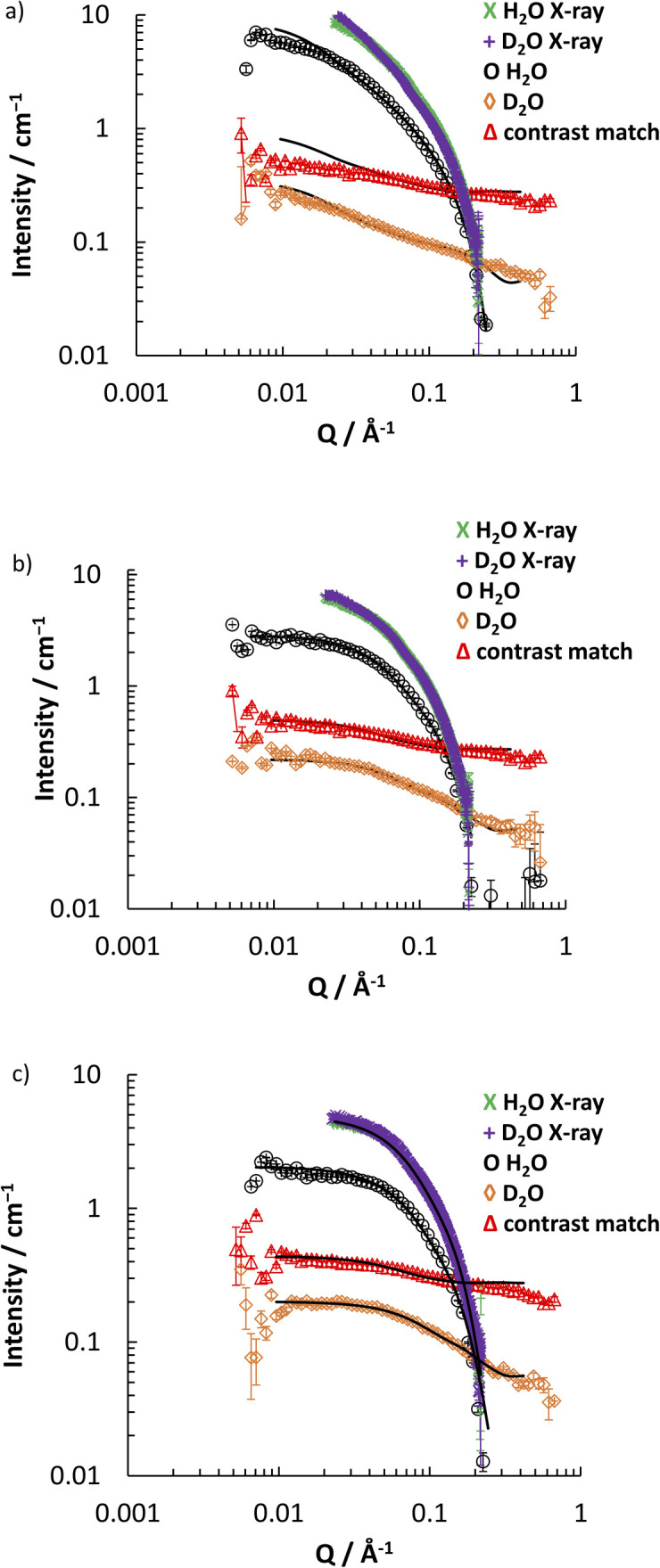
Intensity of small-angle neutron scattering for 100 mmol per dm^3^ magnesium perfluorooctanoate measured at (a) 10 °C (*χ*_R_^2^ = 35) (b) 25 °C (*χ*_R_^2^ = 6.5) and (c) 50 °C (*χ*_R_^2^ = 6) in three different water contrasts. The continuous lines are fits of models for a non-interacting core–shell cylinder with parameters listed in Table 2.

Solutions of 100 mmol per dm^3^ magnesium perfluorooctanoate were investigated with small-angle neutron scattering. The solutions were measured in several water contrasts (100% D_2_O, 64% D_2_O and 100% H_2_O) and at different temperatures (Fig. 5). The data measured with various contrasts were used to determine the hydration and molecular arrangement of the micelles. For Mg^2+^, there was no pronounced peak associated with inter-micellar interactions due to the large screening effect of the magnesium counterion.

At 25 and 50 °C, the X-ray data did not suggest differences due to an isotope effect. As described above, at 10 °C apparently the influence of temperature dominates, and therefore, combined fits of a single model for micelles to all available contrasts were made. As a check of this assumption, a model fit using data only for solutions in D_2_O was made, and this provided adequately consistent results (Fig. 4). The micelles were elongated, maintaining the same core density and number of water molecules in the hydration shell.

The small-angle scattering data were modelled as core–shell cylinders, with co-fitting of complementary small-angle X-ray scattering data. The data were fitted as a mass density of the core, which enabled the scattering length densities of the shell in the different contrasts to be calculated. The core of the micelle consisted of the tail groups and was dense (1.86 ± 0.02 g cm^−3^). The shell consisted of the carboxylate head group with associated water molecules and bound magnesium ions. Approximately 0.4 to 0.45 Mg^2+^ ions per perfluorooctanoate molecule were strongly bound to the shell and less dissociated in solution. Model fits indicated that ∼10 ± 1 water molecules hydrate each headgroup, which was calculated from the volume and scattering length density of the fitted parameters. Varying the degree of counterion decreased the scattering length of the shell in the three contrasts by only 5%, but the amount of water bound to the shell remained unchanged. Previous zeta potential measurements showed a potential of just −18 ± 10 mV for magnesium perfluorooctanoate, which confirms there is less dissociation of the divalent ions as they are more tightly bound to the carboxylate headgroup. A summary of the key parameters for the fitted model is shown in Table 2, and the full fitted parameters for the model are shown in Table S3 in the SI with a comparison of the parameters for fits to only the data measured in D_2_O.

**Table 2:** Summary of key parameters for a core–shell cylinder model for 100 mmol dm^−3^ magnesium perfluorooctanoate at 10, 25 and 50 °C

Temp/°C	Volume fraction	Core radius/Å	Shell thickness/Å	Length/Å	*V*_micelle_/Å^3^	Aggregation no.	No. water per C_7_F_15_COO^−^	Charge per C_7_F_15_COO^−^/e^−^	Core density/g cm^−3^
10	0.049 ± 0.002	11.8 ± 0.2	4 ± 0.1	1500 ± 500	1.2 ± 0.2 × 10^6^	1960 ± 40	10 ± 1	0.1 ± 0.08	1.83 ± 0.02
25	0.049 ± 0.002	12.1 ± 0.2	4 ± 0.1	86 ± 3	73 ± 3 × 10^3^	120 ± 3	10 ± 1	0.1 ± 0.08	1.86 ± 0.02
50	0.049 ± 0.002	11.4 ± 0.2	4 ± 0.1	67 ± 2	53 ± 4 × 10^3^	82 ± 2	10 ± 1	0.1 ± 0.08	1.83 ± 0.02

Our previous studies using small-angle X-ray scattering determined that the micelles were cylinders with an overall length of 82 Å and radius of 14.3 Å.^6^ The fitted parameters combining neutron and X-ray data to fit a model show that at 25 °C the magnesium perfluorooctanoate micelles have a hydrophobic core radius of 12.1 ± 1.0 Å, a shell thickness of 4 Å and a length of 86 Å. Adding moderate polydispersity of about 9 Å, with a log-normal distribution, did not change the fitted average length by more than about 1%.

These values were consistent with those reported previously and confirmed the formation of elongated micelles.^6^ The effect of temperature on the scattering of the magnesium perfluorooctanoate micelles is shown in Fig. 5, with a decrease in intensity as the temperature increased from 10 to 50 °C. For the concentration of 100 mmol dm^−3^, this corresponded to a decrease in aggregation number from 1960 to 81 and a decrease in length from 1500 Å to 67 Å. However, the number of water molecules surrounding the carboxylate group and counterion per surfactant molecule remained the same (Table 2). This showed that the packing of the micelles is maintained despite temperature-dependent changes in size and aggregation.

Uncertainty in the absolute intensity of scattering data is estimated to be approximately 20–25%. For the X-ray measurements, this arises from variations in capillary thickness, leading to transmission and scattering differences of 15–20%, as well as from uncertainties in concentration due to low temperatures outside the solubility limit, where material can precipitate and not fully redissolve. For neutron measurements, contributions arise from uncertainties associated with background subtraction, particularly when measuring with small contrast differences. During fitting, the parameters for the volume fraction and the overall intensity or scale factor are directly correlated, and thus some uncertainty remains when exploiting several independent samples measured with different contrasts or techniques. To verify concentration accuracy and reproducibility, measurements were repeated at 25 °C, and the consistency of the absolute intensity between repeat measurements was confirmed.

The solvated volume of the headgroup is estimated from the fitted data assuming a molecular volume of 30 Å^3^ for water from the H_2_O density of 0.997 g cm^−3^. The carboxylate volume varies between −30 and +15 Å^3^ and increases slightly with temperature. It is apparently dominated by the volume for the Mg^2+^ ion, which has been found to be −60 Å^3^ when hydrated from the determination of the partial specific volume at infinite dilution.^26^ However, the uncertainty arising from the number of water molecules (±1) precludes any detailed interpretation. The negative value arises from the strong hydration when Mg^2+^ binds to water molecules; the structure becomes denser, reducing the volume occupied by the water. The low zeta potential implies that there is only a small degree of dissociation. This could not be determined directly from the scattering experiments, but 0.4 to 0.45 Mg^2+^ ions per carboxylate would be consistent with the data. As with earlier studies of hydrated ions,^26^ the solvated acid headgroup and counterion occupy a smaller partial specific volume than found in crystals and pure liquid acids due to the bonding with the water molecules.^27^

As discussed by others for micelles of ammonium perfluorooctanoate^2^ and sodium perfluorooctanoate,^6^ low-resolution scattering studies cannot readily distinguish between slightly elongated shapes, such as ellipsoids and short rods, with some polydispersity. In the present investigation of magnesium perfluorooctanoate, the radius and shell thickness are reasonably well determined directly from the shape of the scattering curves measured with different contrasts. At lower temperatures, the overall length is constrained by the total intensity of the overall scattering although the measured data do not extend to extremely small values of momentum transfer.

### Comparison of sodium perfluorooctanoate and magnesium perfluorooctanoate micelles

**Fig. 6:**
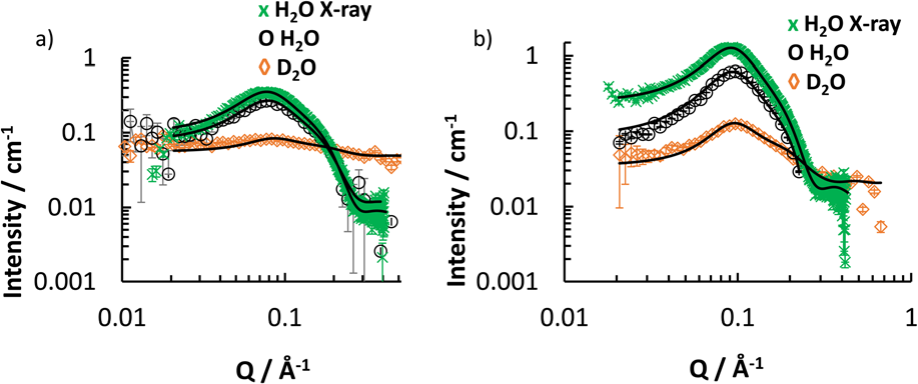
Intensity of small-angle neutron scattering and small-angle X-ray scattering for (a) 100 mmol dm^−3^ (*χ*_R_^2^ = 3) and (b) 200 mmol dm^−3^ (*χ*_R_^2^ = 3) and sodium perfluorooctanoate at 25 °C. The continuous lines are fits of models of a core–shell ellipsoid with the Hayter–Penfold structure factor.

A comparison of the perfluorooctanoate micelles in the presence of Na^+^ and Mg^2+^ counterions revealed variations in the shape and hydration. Scattering data for 100 and 200 mmol per dm^3^ sodium perfluorooctanoate at 25 °C are shown together with the corresponding model fits in Fig. 6. In contrast to magnesium perfluorooctanoate, peaks arising from the interactions were more pronounced in sodium perfluorooctanoate, and the fitted structure factor was determined by the volume fraction of the micelles as well as their charge. The data for samples with the sodium salt were fitted with the Hayter–Penfold rescaled mean spherical approximation as a structure factor that accounted for the screened electrostatic interactions between the micelles.^28–30^ In these models, the interaction was taken as corresponding to the mean shell radius for the ellipsoids.

Sodium perfluorooctanoate micelles were modelled as prolate core–shell ellipsoids because the fitted cylinder length was comparable to that of the micelle diameter. At this aspect ratio, the scattering was not sensitive to the difference between end-capped cylinders and ellipsoids. Previous studies using small-angle scattering^2,6^ also showed that there was no distinction between the cylinders and prolate ellipsoids, and the perfluorooctanoate salts could be fitted using either geometry. The sodium perfluorooctanoate small-angle neutron and X-ray scattering data were co-fitted as described above. In contrast to magnesium perfluorooctanoate, these micelles were smaller, with a radius of 11.8 Å, a thin shell thickness of 3 Å, and a length of 18 Å. The micelles were highly charged with an aggregation number of 30. The core was less dense compared to that of magnesium perfluorooctanoate, with a density of 1.64 ± 0.05 g cm^−3^. The shell contained the carboxylate head group with about 0.2 Na^+^ and about 7 ± 1 water molecules bound per surfactant molecule. The sodium ion was bound less to the carboxylate compared with the magnesium ion, and the high micellar charge indicated greater ionic dissociation. The effect of concentration is also shown in Fig. 6, with an increase in intensity as the concentration increased to 200 mmol dm^−3^. This corresponded to an increase at the higher concentration in micelle length from 18 to 21 Å, an increase in aggregation number from 28 to 36 and an increase in the core density from 1.64 to 1.72 g cm^−3^. The number of water molecules surrounding the carboxylate group and counterion increased from 7 to 9.5. At 300 mmol dm^−3^, the ordered phase behaviour described previously^6^ was observed, making it impossible to fit the data with a simple model.

At 25 °C and a concentration of 100 mmol dm^−3^, the micelles of the magnesium salt had an aspect ratio (length/diameter) of 2.7, which is larger than the ellipticity of 1.6 that was found for the sodium salt. Each carboxylate group was hydrated with 10 water molecules rather than 7; however, the shell was about 1 Å thicker for the magnesium salt. The key parameters for the fitted model are shown in Table 3, and the full list of parameters for the model is shown in Table S4 in the SI. The volume of the water in the headgroup is taken as 30 Å^3^, and the remaining headgroup volume for sodium perfluorooctanoate micelles consisting of the carboxylate ion and 0.2 Na^+^ was calculated as described in the discussion below and found to be 87 Å^3^. This is larger than that found for the magnesium salt, despite the thinner hydrated shell. It reflects the higher degree of ionization as well as the small partial specific molecular volume for a hydrated Na^+^ ion at infinite dilution of 11 Å^3^.^26^

**Table 3:** Summary of key parameters for a core–shell ellipsoid model for 100 and 200 mmol dm^−3^ sodium perfluorooctanoate at 25 °C

Conc./mmol dm^−3^	Volume fraction	Equatorial radius/Å	Shell thickness/Å	Polar radius/Å	*V*_micelle_/Å^3^	Aggregation no.	No. water per C_7_F_15_COO^−^	Charge per C_7_F_15_COO^−^/e^−^	Core density/g cm^−3^
100	0.021 ± 0.001	11.8 ± 0.2	3.0 ± 0.2	18 ± 2	19 ± 3 × 10^3^	28 ± 2	7.0 ± 0.5	0.8 ± 0.1	1.64 ± 0.05
200	0.051 ± 0.002	12.1 ± 0.1	3.1 ± 0.2	21 ± 1	23 ± 2 × 10^3^	36 ± 2	9.5 ± 0.5	0.8 ± 0.1	1.72 ± 0.03

**Fig. 7:**
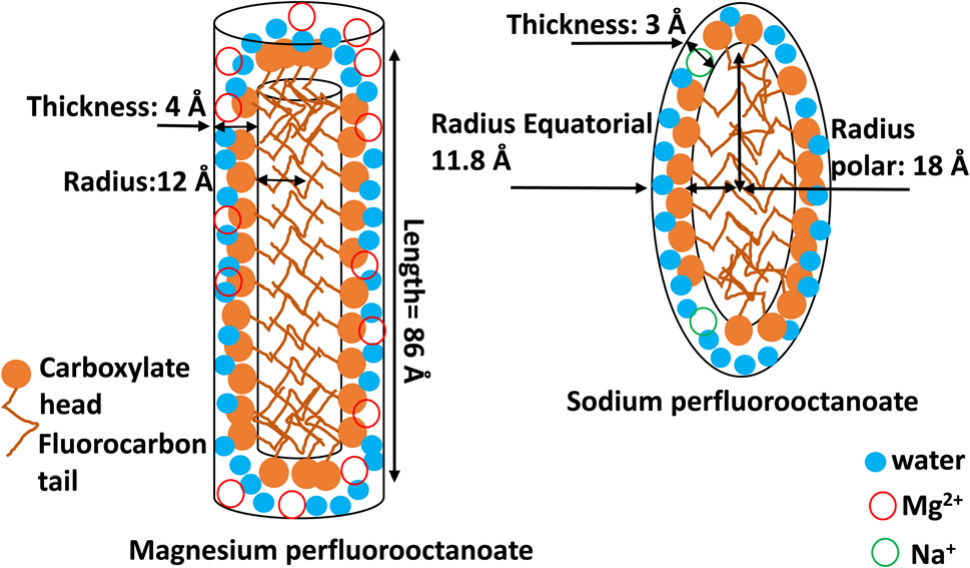
Schematic illustration of the core–shell cylinder and core–shell prolate ellipsoid for a micelle of magnesium perfluorooctanoate and sodium perfluorooctanoate, respectively. The number of water molecules per headgroup is, in reality, much higher than can be represented in a planar cut.

A schematic illustration comparing the micelles of sodium and magnesium perfluorooctanoate is shown in Fig. 7. The magnesium perfluorooctanoate micelles were about five times longer and had more water in the shell. The divalent ion influences the core density, thus modifying the packing, causing molecules to pack more densely in less curved assemblies.

### Structural and thermodynamic implications

Hydrogen isotope effects were reported previously in ammonium perfluorooctanoate systems.^22^ These systems were studied at higher concentrations and temperatures than those in the present study. The authors suggested that the micelles in D_2_O were slightly smaller than those in H_2_O, with a temperature difference of 2 K between the liquid crystal phase boundaries when substituting D_2_O with H_2_O. These effects were attributed to the interactions between perfluorooctanoate micelles, together with changes in the packing of the molecules, contributed to tighter binding of the ammonium ions to the surfactant head group in the D_2_O system. Different isotope effects were observed in the CsPFO/H_2_O and CsPFO/D_2_O systems, with the bigger micelles observed in D_2_O.^23^ The latter study suggested that D_2_O forms stronger hydrogen bonds compared to H_2_O and also suggests that the Cs^+^ was tightly bound to the carboxylate ion; substituting D_2_O with H_2_O resulted in a stronger bond, providing more effective Cs^+^ screening.^23^ The differences in hydrogen bonding in D_2_O and H_2_O have been attributed to the differences in bond lengths between deuterium and oxygen compared to hydrogen and oxygen. In particular, deuterium forms hydrogen bonds that are longer and stronger, leading to differences in size and packing.^24,25^ For the present system, it is therefore likely that differences in bond length and hydrogen bond strength between the carboxylate ion and hydrated ion contribute to the observed size discrepancy, particularly close to temperatures where there are relatively large differences in the properties of H_2_O and D_2_O. It is interesting that no large systematic isotope effects were seen in the earlier study using several different mixtures of H_2_O and D_2_O for ammonium perfluorooctanoate with samples that were buffered at pH 8.8.^2^

Nuclear magnetic resonance (NMR) studies on the micellization of ammonium and tetra-alkylammonium perfluorooctanoates showed that for the carbon groups in the core, the chemical shifts with aggregation number were relatively close, suggesting that the chemical environment was almost the same. This was consistent with the present study because the core density was constant. For the carbon group next to the head carboxyl group, the chemical shifts were smaller because it has some contact with the aqueous phase. This low shift is explained as arising because the head group is very hydrated and has a bound counterion.^31^ Similar observations were seen in NMR studies on caesium perfluorooctanoate.^32^ Isotope effects could be detected in such NMR studies if they were performed in D_2_O, as the different solvent environment would show the change in hydrogen bonding between the two water contrasts.

The present study determined the core density of the magnesium perfluorooctanoate to be 1.86 g cm^−3^, corresponding to a volume of 331 Å^3^ for the perfluoroheptyl moiety. The corresponding values for sodium perfluorooctanoate were 1.64 g cm^−3^ and 373 Å^3^, respectively. The lower core density of the micelles of the sodium perfluorooctanoate salt compared to the magnesium salt suggests that the molecular packing adopts a less-ordered assembly driven by the repulsion of the carboxylate groups, which are not ‘neutralized’ with the counterions in close proximity. Previous small-angle neutron scattering studies reported densities of 1.98 g cm^−3^ for a perfluorooctanoate ion corresponding to a volume of 346 Å^3^,^2^ and 1.84 g cm^−3^ that corresponds to a volume of 334 Å^3^ for the moiety C_7_F_15_.^3^ It is interesting that these values for the core density are all slightly higher than those that are found for short liquid fluoroalkanes (C_6_F_14_, C_7_F_16_ and C_8_F_18_) at 25 °C,^33^ which would give an estimate for the perfluoroheptyl volume of 326 Å^3^. This indicates that the packing in the core of the micelle is likely to be more ordered and slightly denser than that of a liquid but far below that expected for crystalline fluoroalkanes. The number of water molecules in the shell of sodium perfluorooctanoate was reported to be ∼8, matching the value of ∼7 water molecules determined in the present study. There is a slight tendency that is apparent for a higher micellar core density as the concentration increases. This could not be explored in more detail as an ordered liquid crystalline phase appears with sharp structural peaks.

## Conclusions

The present study compares how magnesium ions and sodium ions influence the structure and hydration of perfluorooctanoate micelles. In the presence of Mg^2+^, the shell of the perfluorooctanoate micelles was highly hydrated with ∼10 water molecules per carboxylate, consistent with previous NMR observations. Magnesium salts are more hydrated, with headgroup packing influenced by the partial specific volumes of the solvated ions. This alters the packing parameter, resulting in micelles with denser fluorocarbon cores in the less curved assemblies. The magnesium and sodium ions influence the core density; the core density increases from 1.64 to 1.86 g cm^−3^ as the counterion changed from Na^+^ to Mg^2+^. Magnesium perfluorooctanoate micelles formed elongated cylinders, in contrast to the sodium perfluorooctanoate micelles, which were only slightly elliptical.

The effect of temperature was seen as the length of the micelles decreased from 1500 to 67 Å with increasing temperature, but the core density and the shell hydration remained the same.

An isotope effect is observed at low temperatures and when D_2_O is exchanged for H_2_O. At 15 °C, the perfluorooctanoate micelles were 8 times longer in D_2_O than in H_2_O. This effect is likely to be of considerable significance for investigations, such as NMR studies, of the perfluorooctanoate micelles studied here and similar materials when performed in heavy water. The addition of extra magnesium chloride decreased the solubility of the magnesium perfluorooctanoate, with the surfactant precipitating from the solution. Heating the samples to 50 °C did not increase the solubility. Careful constrained fitting was necessary for reliable interpretation of the structure factor and form factor for sodium perfluorooctanoate.

With respect to the natural environment, the ionic composition of natural waters such as lakes and seawater is relevant. The ionic composition of freshwater contains about 0.249 mmol per dm^3^ Na^+^ and 0.060 mmol per dm^3^ Mg^2+^,^34^ while seawater contains much higher levels of 470 mmol kg^−1^ and 53 mmol kg^−1^, respectively.^35^ In environments contaminated with perfluorocarbon surfactants, a potential treatment approach is to concentrate the contaminated water until there is sufficient salt to induce surfactant precipitation. Such salt-driven precipitation can provide the first stage of potential future remediation methods by separating and concentrating the surfactants before degrading them through processes such as electrochemical oxidation and plasma treatment, both of which lead to defluorination. For example, cerium dioxide (CeO_2_) electrodes enhanced with oxygen have been used for the defluorination of perfluorooctanoic acid.^36^ Plasma-based water treatment has been applied to perfluorooctanoic acid in water, resulting in defluorination.^37^

## Author contributions

Njelama Sanga writing – original draft, investigation, methodology, formal analysis, conceptualization, data curation. Lutz Ahrens writing – review and editing, supervision, project administration, conceptualization, methodology. Gregory N. Smith writing – review and editing, investigation, methodology, data curation. Adrian R. Rennie writing – review and editing, conceptualization, funding acquisition, project administration, supervision, formal analysis, data curation, methodology.

## Conflicts of interest

There are no conflicts of interest to declare.

## Supplementary Material

RA-OLF-D6RA02082H-s001

## Data Availability

Neutron scattering data will be available with DOI: https://doi.org/10.5286/ISIS.E.RB2420153. Other data are available on reasonable request. Supplementary information (SI): details of the solution preparation, a description of the strategy to produce the structural models, and tables of fitted parameters. Background information with tables of literature values for the volumes suggested for perfluorooctanoate in micelles and expressions for *χ*^2^ and reduced *χ*_R_^2^. Additional plots of the data as referenced in the text are shown. See DOI: https://doi.org/10.1039/d6ra02082h.
