# Supplementary material for: Influence of magnesium on the structure and hydration of perfluorooctanoate micelles: comparison with sodium salt
Source: RSC Adv. 2026 Aug 13;16(40):47235–45. doi: 10.1039/d6ra02082h (PMC13470671; doi:10.1039/d6ra02082h)
Supplement: Supplementary file 1 [file RA-016-D6RA02082H-s001.pdf]

Supplementary Information (SI) for RSC Advances.
This journal is © The Royal Society of Chemistry 2026

Supplementary Information

Influence of magnesium on the structure and hydration of perfluorooctanoate micelles – Comparison with sodium salt

Njelama Sanga[1*], Lutz Ahrens[2], Gregory N. Smith[3], Adrian R. Rennie[1]

[1] Macromolecular Chemistry, Department of Chemistry - Ångström, Uppsala University, Box 539, 75120 Uppsala, Sweden

[2] Department of Aquatic Sciences and Assessment, Swedish University of Agricultural Sciences (SLU), Box 7050, 75007 Uppsala, Sweden

[3] ISIS Neutron and Muon Source, Science and Technology Facilities Council, Rutherford Appleton Laboratory, Didcot OX11 0QX, United Kingdom

*njelama.sanga@kemi.uu.se

1. Preparation of solutions

The magnesium perfluorooctanoate stock solution was prepared at 150 mmol dm^{-3} by directly dissolving the powder in three different water contrasts H_2O, 64%$_{vol}$ D_2O, D_2O. Dilutions of the 150 mmol dm^{-3} stock solution in H_2O to 100 mmol dm^{-3}, 75 mmol $dm^{-3}$and 50 mmol dm^{-3}, respectively, were prepared by weight. The stock solutions of sodium perfluorooctanoate were prepared at 300 mmol dm^{-3} in the same water contrasts as above and diluted to 200 mmol dm^{-3}, 150 mmol dm^{-3} and 100 mmol dm^{-3}. Both surfactant solutions had pH 8 measured with pH paper. The magnesium perfluorooctanoate solutions prepared for small-angle X-ray scattering were made in the same way but at 100 mmol dm^{-3} and in two different water contrasts H_2O and D_2O.

2. Model fitting

It is helpful to clarify how the structural model for micelles was established and the composition of the core and shell regions of micelles from the fitted parameters.

1) The core of the micelle contains the fluoroalkyl groups C_7F_{15} only. This was established as fits were not improved by allowing the scattering length density of the core to vary for the measurements with different composition of the water with respect to H_2O and D_2O. This clarifies that the region modelled as 'core' does not contain water or hydrated ionic groups.

2) A constraint was applied during fitting to relate the fitted neutron scattering length density of the core to that for X-rays assuming an empirical chemical formula of C_7F_{15} that corresponds to dry fluorocarbon material by multiplying by the ratio of the scattering lengths that are well established for carbon and fluorine atoms of:

   $b_{core\_X\text{-}rays} = b_{core\_neutrons} \times 3.75$ Equation S1.

   This can be related to the mass density, ρ_m of the core by:

   $\rho_m / \rho_{core\_neutrons} = 4.67$ ng /m Equation S2.

3) The scattering length densities of the core and shell of the micelles were fitted and used to calculate derived micellar parameters N_{agg} and N_w using equations (1) and (2) in the main text and the scheme provided in Figure 1 and these values are shown in Table S3 and S4.

4) The shell consists of the hydrated carboxylate group (COO^-) and hydrated counterion (Mg^{2+}/ Na^+), and water. The product of the shell volume, V_s, and the shell scattering length density, ρ_s, divided by the micellar aggregation number provides a total scattering length per fluorocarbon for the hydrated head groups, associated cation fraction and water within the micelle, b_s.

   $b_s / N_{agg} = (V_s \rho_s / N_{agg}) - N_w b_w$ Equation S3.

   Assuming stoichiometry, there is one carboxylate per fluorocarbon, a fraction, f of the cation and N_w water molecules with scattering length b_w per head group. This was calculated for each contrast used in model fitting and the model was rejected if it did not provide a consistent value for N_w for all measured contrasts.

5) The fraction of cations is determined directly from the ionic dissociation derived from the fits to the data for the sodium salt. For example, an electrostatic charge of 0.8 e^- per carboxylate for the NaPFO would correspond to 0.2 Na^+ per carboxylate remaining tightly associated in the shell region of the micelle. An electrostatic charge of 0.1 e^- per carboxylate for the MgPFO would correspond to 0.4 Mg^{2+} per carboxylate remaining tightly associated in the

shell region of the micelle. This is described in the main text under the section describing magnesium perfluorooctanoate - micellar shape and dimensions.

6) The volume of each molecule of water is always approximated as 30 Å^3. This permits estimates of the partial specific volume associated with the hydrated ionic groups.

7) For dilute systems the intensity of the scattering is proportional to the volume fraction, ϕ_{mic}, of micelles. In models with no significant contribution from a structure factor that describes inter-micellar correlations, this is represented by the scale factor in the SasView software. The fraction of surfactant in the samples that is freely dissolved, given by the critical micelle concentration, C_{cmc}, can be significant and so this is calculated from the total sample concentration, C_{tot}, as

$$\phi_{mic} = (C_{tot} - C_{cmc}) / \rho_{mic} \qquad \text{Equation S4}$$

where ρ_{mic} is the mass density of the micelles found from measurements of density of dispersions. When micelles are modelled with strong interactions, the volume fraction is a parameter used explicitly in the calculation of the structure factor and in that case the separate 'scale factor' is maintained at 1.

8) The scattering length for each moiety is as shown in Table S1.

3. Literature values of volumes suggested for perfluorooctanoate in micelles

Table S1. Comparison of the molecular volumes and scattering lengths of the moieties reported in literature for fitting SANS data of perfluorooctanoate salts

Moiety	Vol. / Å^3	Reference for volume	Neutron scattering length* / fm
CF_2	44.9	1	17.96
	41.6	2	17.95
CF_3	73.7	1	23.61
	84	2	23.61
COO^-	37	1	18.26
	47.4	2	18.25
H_2O	29.9	1	-1.67
	29.9	2	-1.67
D_2O	29.9	1	19.15
	30.19	2	19.14
$CF_3(CF_2)_6$	333.6	2	131.3
	343.1	3	
$CF_3(CF_2)_6COO^-$	380.1	1	149.6
	381	2	
	346	3	

*Calculated from the tabulated values of atomic scattering lengths compiled by V. F. Sears (4).

4. Expressions for chi squared, χ^2 and reduced chi squared, χ_R^2

Curve fitting of models to measured data was made by minimising the reduced chi-squared parameter, χ_R^2. χ^2 is the sum of the squared residuals (difference between model, I_{model}, and measured data) divided by the square of a weighting factor, w_i. which is taken as the experimental uncertainty for each measured value, I_{meas}, of the intensity. This quantity is dimensionless and independent of units and is expressed as:

$$\chi^2 = \sum_i\left(\left(I_{\mathrm{meas},i} - I_{\mathrm{model},i}\right)^2/(w_i^2)\right) \qquad \text{Equation S5.}$$

The overall goodness of the fit is assessed with the reduced chi-squared parameter, χ_R^2, which, is also dimensionless and independent of units, but is normalized by the 'degrees of freedom'. This is the number of data points, N_{pts}, minus the number of fitted parameters, N_{par}:

$$\chi_R^2 = \sum_i\left(\left(I_{\mathrm{meas},i} - I_{\mathrm{model},i}\right)^2/(w_i^2)\right) / (N_{pts} - N_{par}) \qquad \text{Equation S6}$$

and provides a normalised statistic that would approximate unity if the deviations of the model match the experimental uncertainty. This would be independent of both the choice of units and the number of data points. In the model fitting described in this work, χ_R^2 is calculated for all data with the various contrasts that are included. The minimisation was made with parameters for the different neutron and X-ray contrasts constrained as described in section S2 above to all data for a given concentration of surfactant simultaneously.

5. Model fit Parameters

Table S2. Fits to individual SAXS data sets in H_2O and D_2O for 100 mmol dm^{-3} magnesium perfluorooctanoate to a model of uniform cylinders

Temperature / °C	Radius H_2O / Å ± 0.1	Radius D_2O / Å ± 0.1	Length H_2O / Å	Length D_2O / Å	Volume H_2O / Å^3	Volume D_2O/ Å^3	Aggregation no. H_2O	Aggregation no. D_2O
10	14.1	14.4	1980 ± 500	1900 ± 500	$1.2 \pm 0.3 \times 10^6$	$1.25 \pm 0.3 \times 10^6$	1600	1600
15	14.3	14.1	179 ± 5	1510 ± 500	$0.11 \pm 0.03 \times 10^6$	$0.94 \pm 0.3 \times 10^6$	150	1200
25	14.5	14.4	90 ± 10	100 ± 2	$59 \pm 6 \times 10^3$	$65 \pm 2 \times 10^3$	75	83
50	14.2	14.2	72 ± 3	75 ± 3	$46 \pm 2 \times 10^3$	$48 \pm 2 \times 10^3$	59	61
25 repeated	14.5	14.3	89 ± 10	101 ± 2	59 ± 6 x 10^3	66 ± 2 x 10^3	77	87

Note: To fit the X-ray scattering data, the measured background was subtracted from the data, and hence this was fixed at 0 for both contrasts in the model. The volume fraction for both water contrasts was fitted and within the range of 0.04 ± 0.002. The scattering length density for the surfactant and solvent were fixed at 14.8×10^{-6} Å^{-2} for the surfactant and 9.44×10^{-6} Å^{-2} for the H_2O and D_2O solvents. The aggregation numbers were calculated using the scattering length of the molecule.

Table S3. Full Fitted parameters for a core-shell cylinder model at different temperatures for 100 mmol dm^{-3} magnesium perfluorooctanoate using multiple contrasts

Temperature / °C	Volume fraction	Core Radius / Å (C_7F_{15})	Shell Thickness / Å	Length* / Å	SLD_{core} / 10^{-6} Å^{-2} (C_7F_{15}) Neutron	SLD_{core} / 10^{-6} Å^{-2} (C_7F_{15}) X-ray	V_{core} / Å^3	$V_{micelle}$ / 10^6Å^3
10	0.049	11.8 ± 0.2	4	1500 ± 500	4.01 ± 0.05	15.1 ± 0.1	66 ± 2 x10^4	1 2 ± 0.3
15	-	-		-	-	-	-	-
25	0.049	12.1 ± 0.2	4	86± 3	4.07 ± 0.04	15.3 ± 0.5	40 ± 3 x 10^3	0.073 ± 0.002
50	0.049	12.6 ± 0.2	4	67 ± 2	4.00 ± 0.05	15.02 ± 0.2	27 ± 2 x 10^3	0.053 ± 0.002
Fit only D_2O								
10	0.049	12.3 ± 0.2	4	2000± 500	4.00 ± 0.04	15.0 ± 0.1	94 ± 1 x 10^4	1.7± 0.2
15	0.049	11.8 ± 0.2	4	1400± 500	4.08 ± 0.05	15.3 ± 0.1	65 ± 3 x 10^4	1.15 ± 0.1

Temperature / °C	Background D_2O / cm^{-1}	Background H_2O / cm^{-1}	Background 64% D_2O / cm^{-1}	$SLD_{shellD2O}$ / 10^{-6} Å^{-2}	$SLD_{shellH2O}$ / 10^{-6} Å^{-2}	$SLD_{shellL64\% D2O}$ 10^{-6} Å^{-2}	$SLD_{shellD2O}$ / 10^{-6} Å^{-2} X-ray	$SLD_{shellH2O}$ / 10^{-6} Å^{-2} X-ray
10	0.043 ± 0.005	0	0.27 ± 0.01	8.1 ± 0.2	0.014	5.65	11.9 ± 0.3	12.1 ± 0.1
15	-	-	-	-	-	-	-	-
25	0.050 ± 0.002	0.0116	0.27 ± 0.01	7.4 ± 0.3	0.19	5.54	12.1 ± 0. 3	12.0 ± 0.2
50	0.056 ± 0.002	0	0.27 ± 0.01	7.2 ± 0.2	0.45	5.51	12.6 ± 0. 4	12.3 ± 0.2
Fit only D_2O								
10	0.041 ± 0.002	-	-	8.2 ± 0.1	-	-	11.7 ± 0.3	-
15	0.043 ± 0.001	-	-	7.9 ± 0.2	-	-	11.9 ± 0.1	-

*The sensitivity of the cylinder model to the length of the micelles is illustrated in Figure S4. As only the points at lowest Q are sensitive to the longest lengths, the relative uncertainty in the fitted length increases as shown.

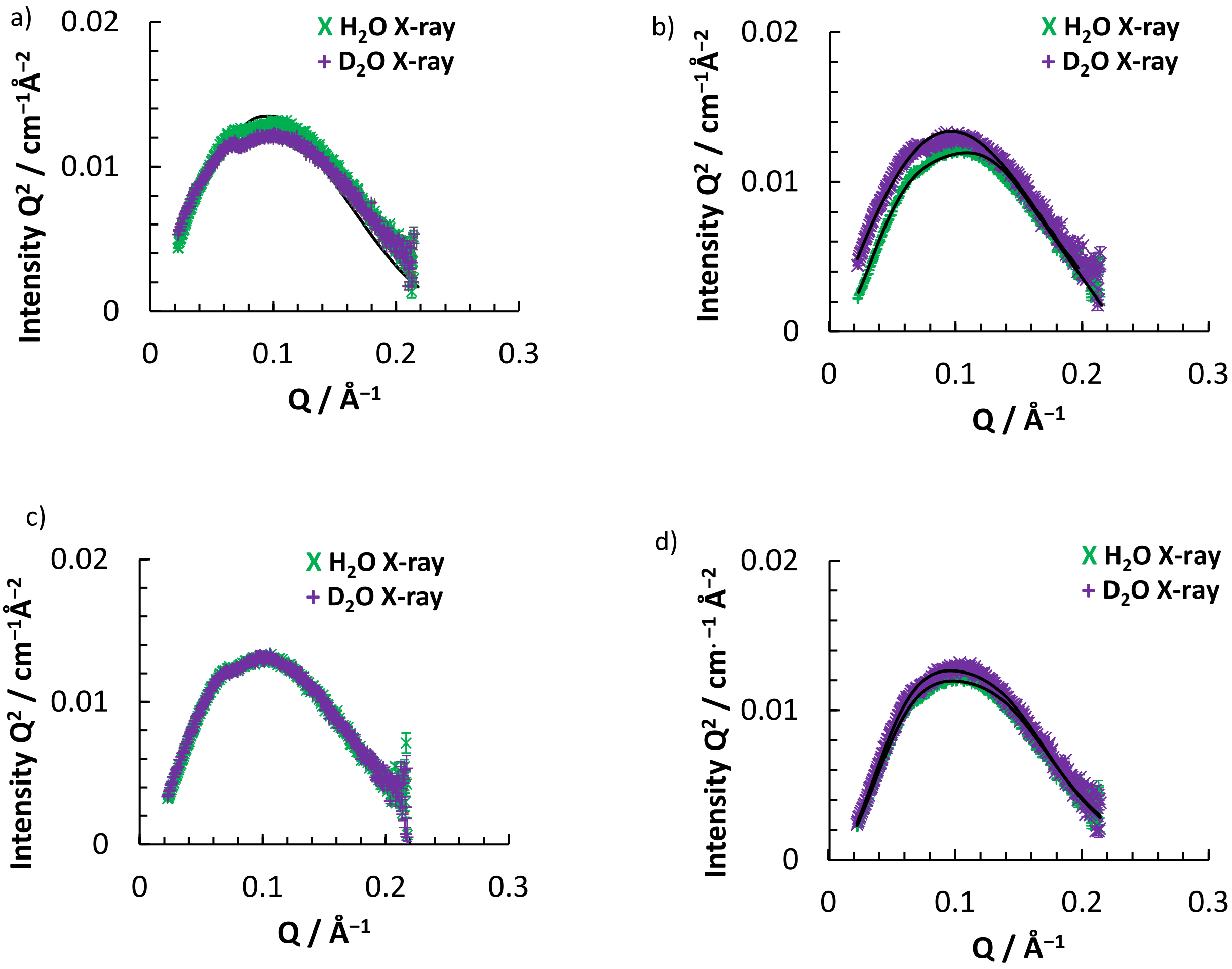

Figure S1. Kratky plots (I Q^2 vs Q) of small-angle X-ray scattering data for 100 mmol dm^{-3} magnesium perfluorooctanoate measured at a) 10°C b) 15°C c) 25°C and d) 50°C in D_2O and H_2O. The continuous lines represent fits of models for a cylinder.

Table S4. Full Fitted parameters for models of micelles for 100 and 200 mmol dm^{-3} sodium perfluorooctanoate at 25 °C

Conc. / mmol dm^{-3}	Volume fraction	Equatorial radius / Å	Shell Thickness / Å	Ellipticity	Polar Radius / Å	SLD_{core}/ 10^{-6} Å^{-2} (C_7F_{15}) Neutrons	SLD_{core} / 10^{-6} Å^{-2} (C_7F_{15}) X-ray	V_{core} / 10^3Å^3	$V_{micelle}$ / 10^3 Å^3
100	0.021	11.8	3 ± 0.2	1.56 ± 0.02	18 ± 1	3.58 ± 0.03	13.46 ± 0.10	10.6± 0.2	19.4 ± 0.4
200	0.051	12.1	3.1 ± 0.2	1.73 ± 0.04	21 ± 1	3.77 ± 0.03	14.15 ± 0.14	12.9 ± 0.3	23 ± 1
Conc. / mmol dm^{-3}	$Background_{D2O}$ / cm^{-1} Neutron	$Background_{H2O}$ / cm^{-1} Neutron	$Background_{H2O}$ / cm^{-1} X-ray	$SLD_{shellD2O}$ / 10^{-6} Å^{-2} Neutron	$SLD_{shellH2O}$ / 10^{-6} Å^{-2} Neutron	SLD_{H2O} / 10^{-6} Å^{-2} X-ray	Charge per $C_7F_{15}COO^-$ / e^-	Aggregation no.	Core Density / g cm^{-3}
100	0.046 ± 0.001	0.0031 ± 0.0001	0.0075 ± 0.0004	7.55 ± 0.02	0.60 ± 0.10	11.75 ± 0.02	0.8	28 ± 2	1.64 ± 0.05
200	0.020 ± 0.001	-0.022 ± 0.0001	0.012 ± 0.0001	7.39 ± 0.02	0.11± 0.02	12.23 ± 0.01	0.8	36 ± 2	1.72 ± 0.03

6. Extra data

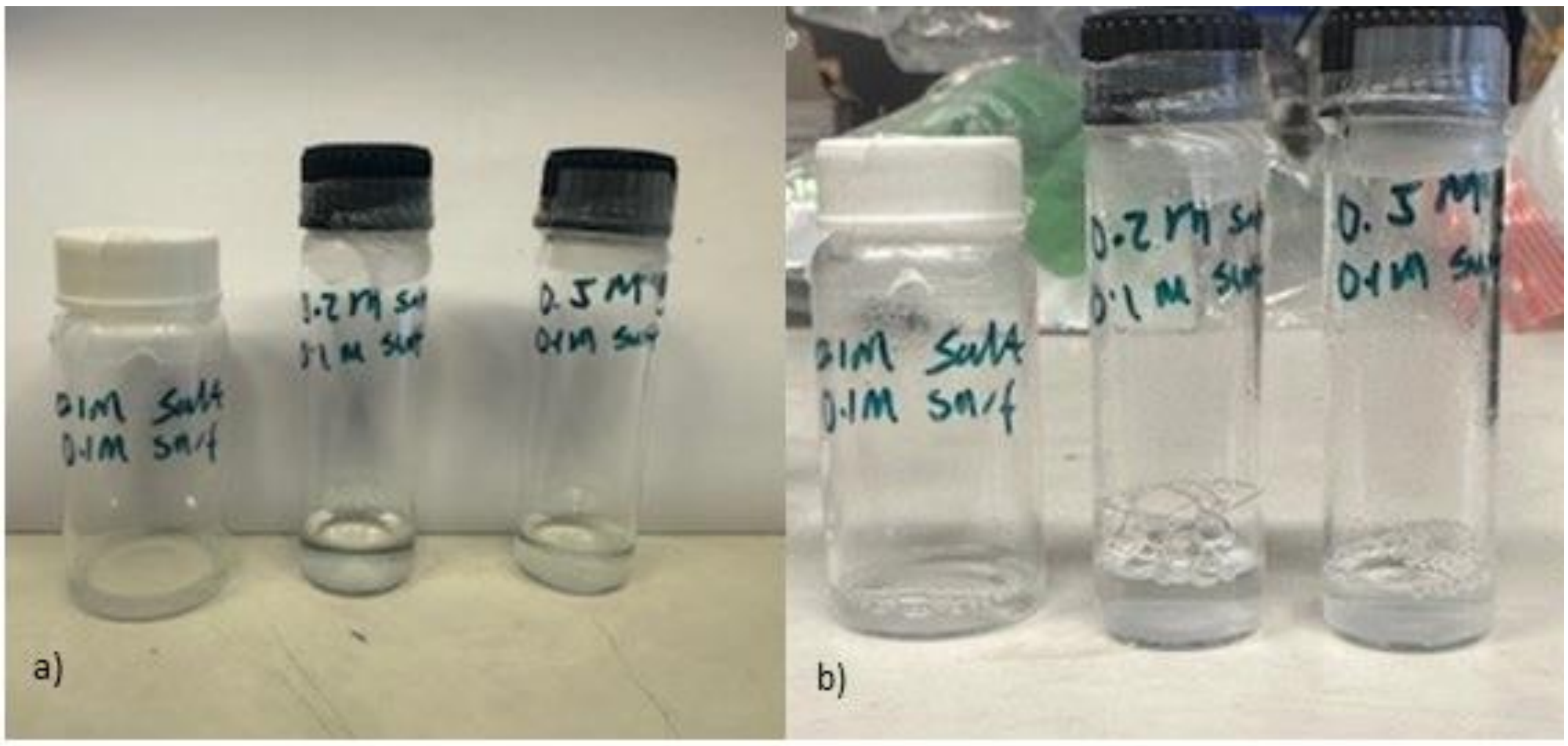

Figure S2. Photograph showing the influence of added magnesium chloride hexahydrate at 0.1, 0.2 and 0.5 mol dm^{-3} on solutions of 100 mmol dm^{-3} magnesium perfluorooctanoate at a) 22°C and b) 50°C. At both temperatures the surfactant remains as a precipitate.

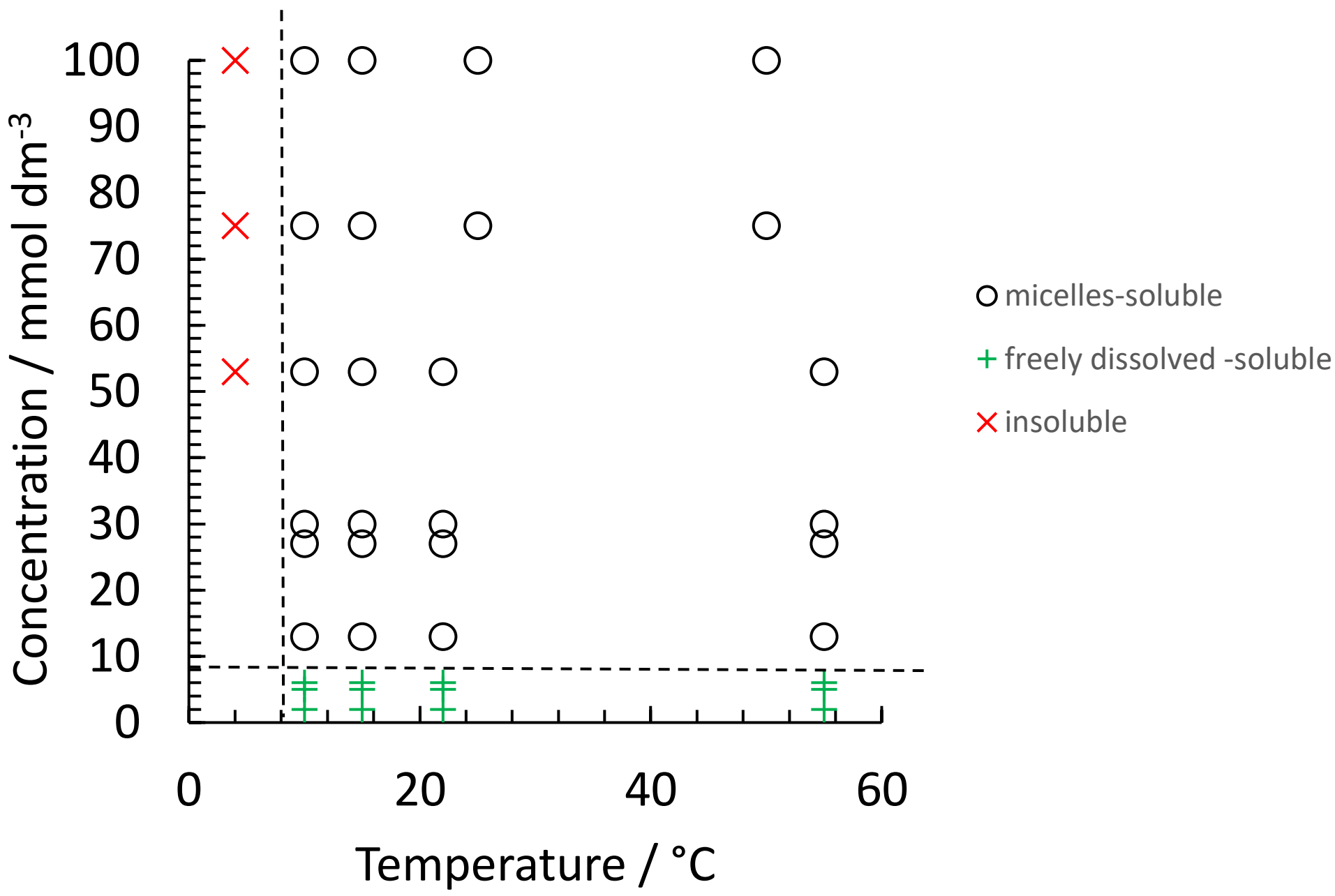

Figure S3. Phase diagram for the magnesium perfluorooctanoate / H_2O system as a function of concentration vs temperature. The horizontal dashed line indicates the critical micelle concentration that does not vary greatly with temperature, below this line the ions are freely dissolved, above this line micellization begins. The vertical line intercepting the temperature axis indicates the solubility limit or Krafft boundary. Insufficient data are available to provide corresponding phase maps as the concentration of added salt was varied.

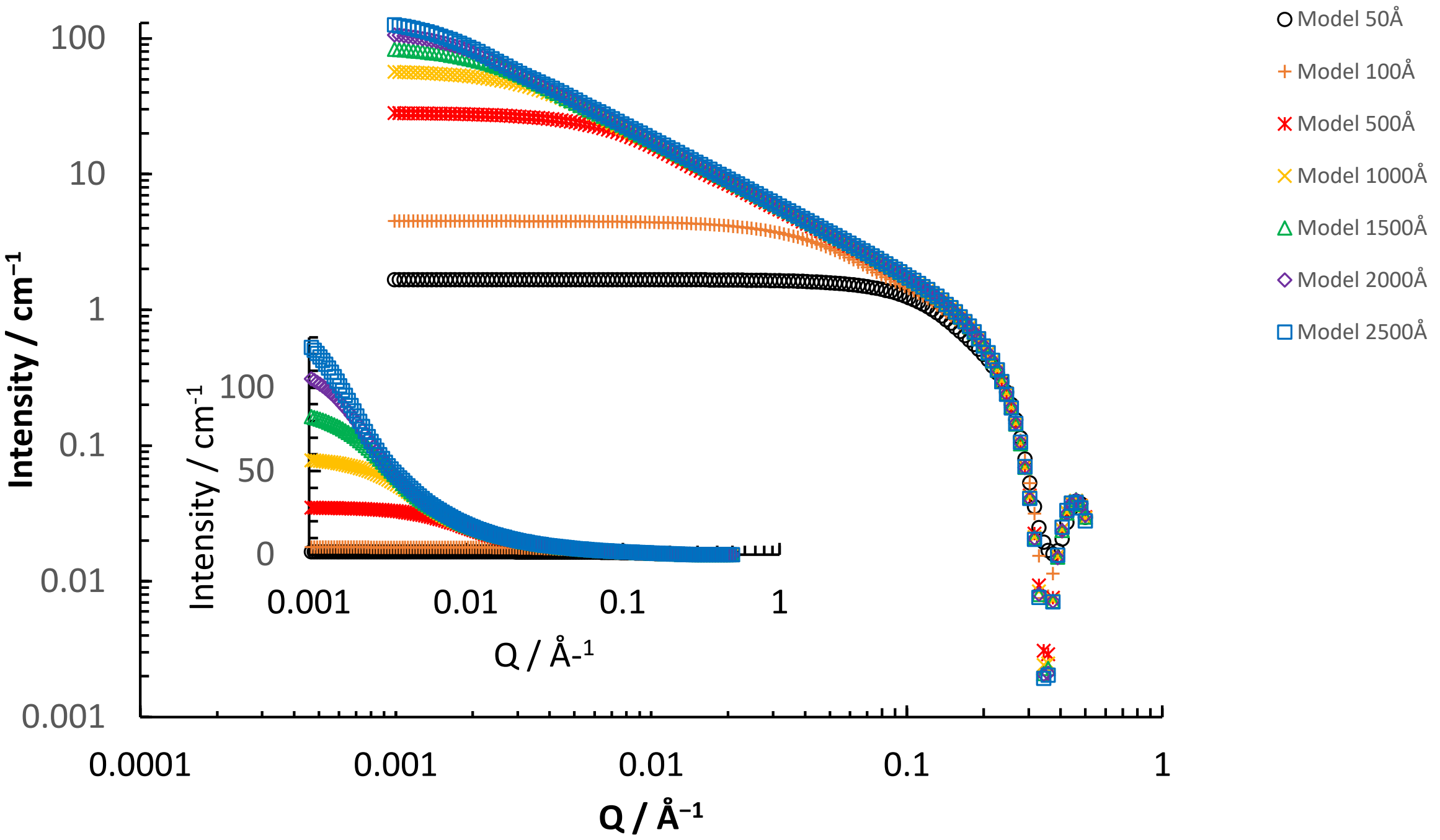

Figure S4. Model scattering curves calculated using a core-shell cylinder model in D_2O for micelle lengths of 50, 100, 500, 1000, 1500, 2000, and 2500 Å with identical values for the radius (12 Å), shell thickness (4 Å), and the core (4×10^{-6} Å^{-2}), shell (7.4×10^{-6} Å^{-2}) and solvent (6.35×10^{-6} Å^{-2}) scattering length densities. At high Q, all curves overlap, reflecting the scattering from the cylinder cross section. At low Q, the curves diverge with increasing micelle length, showing the sensitivity of the low Q scattering to the overall micelle length. The inset shows an expanded region at low Q.

References

1) Berr, S. S.; Jones, R. R. M. Small-angle neutron scattering from Aqueous Solutions of Sodium Perfluorooctanoate above the Critical Micelle Concentration, *J. Phys. Chem.,* **1989**, 93, 2555-2558.
2) Kancharla, S.; Jahan, R.; Bedrov, D., Tsianou, M.; Alexandridis, P. Structure and Interactions in Perfluorooctanoate Micellar Solutions Revealed by Small-angle Neutron Scattering and Molecular Dynamic Simulations Studies: Effects of Urea, *Langmuir*, **2021**, 37, 5339-5347.
3) Burkitt, S. J.; Ottewill, R. H.; Hayter, J. B.; Ingram, B. T. Small-angle Neutron Scattering Studies on Micellar Systems Part. 1. Ammonium Octanoate, Ammonium Decanoate and Ammonium Perfluorooctanoate. *Colloid & Polym. Sci.,* **1987**, 265, 619-627.
4) Sears V. F. Neutron scattering lengths and cross sections *Neutron News*, **1992**, 3(3), 26-37.
